# Mapping the research landscape and evolving hotspots of intensive care unit-acquired weakness: a dual-database bibliometric analysis

**DOI:** 10.3389/fneur.2026.1759594

**Published:** 2026-04-08

**Authors:** Xuehua He, Peiqi Liu, Xiaoyan Gong, Yiyu Zhuang, Xiangping Chen

**Affiliations:** Nursing Department, Sir Run Run Shaw Hospital, Zhejiang University School of Medicine, Hangzhou, China

**Keywords:** bibliometric, critical care medicine, intensive care unit-acquired weakness, nursing, rehabilitation

## Abstract

**Purpose:**

This review aimed to summarize and map the existing literature to clarify the temporal and spatial distribution, interconnections, and emerging trends in Intensive care unit-acquired weakness (ICU-AW) research.

**Patients and methods:**

Bibliographic records were retrieved from the Web of Science Core Collection (WoSCC) and PubMed database. VOSviewer and CiteSpace were used to visualize the publication landscape, analyze citation patterns and collaborative networks, and conduct cluster analysis, burst keyword detection, and timeline mapping.

**Results:**

A total of 1,449 publications from WoSCC and 648 publications from PubMed were included in analysis, representing contributions from 72 countries, 6,908 authors, and 365 journals. In recent years, authors from Asian countries and journals focusing on clinical nutrition have shown rapid growth. We identified 11 thematic clusters and 67 burst keywords from WoSCC publications, indicating a shift in research focus toward early identification, early functional rehabilitation, and nutritional support. External validation of the research topics and trends in PubMed database yielded results consistent with the preliminary analysis.

**Conclusion:**

Over the past two decades, research on ICU-AW has expanded steadily, with increasing contributions from East Asia and nutrition- and neuromuscular-focused journals, while Europe, North America, and critical care publications continue to dominate. A total of 11 major research clusters were identified, encompassing the diagnosis, prevention, treatment, and prognosis of ICU-AW. These findings map the evolution of research themes and may help identify emerging directions and generate hypotheses for future studies.

## Introduction

With the global rise in critical illness and rapid advancements in intensive care medicine (1), the mortality rate among critically ill patients has steadily declined (2). Consequently, more patients now survive episodes of critical illness after treatment in the intensive care unit (ICU). However, this improvement in survival has been accompanied by a marked increase in ICU-related complications.

Intensive care unit-acquired weakness (ICU-AW) is a syndrome characterized by generalized limb weakness that develops during ICU stay without other identifiable causes (3). It primarily presents as symmetrical muscle weakness and is associated with multifactorial pathophysiological mechanisms involving both muscle mass and nerve function (3). Existing evidence indicates that up to 48% of patients in the ICU develop ICU-AW (4), which is associated with profound and long-lasting adverse effects on clinical outcomes and physical recovery. ICU-AW negatively affects short-term outcomes of patients during ICU management, including failure of ventilator weaning, prolonged dependence on mechanical ventilation, extended ICU length of stay, and increased mortality (5). Furthermore, its detrimental impact on physical function and health-related quality of life can persist for 6 to 18 months after hospital discharge (6, 7), imposing significant long-term burdens on patient rehabilitation and overall prognosis.

Given its clinical significance, ICU-AW has drawn increasing attention from multiple disciplines, including critical care medicine, nursing, rehabilitation, and clinical nutrition. Several narrative reviews and systematic reviews have summarized the pathophysiology, risk factors, diagnostic approaches, and management strategies of ICU-AW (8–10). However, these studies primarily focus on clinical evidence synthesis and do not provide a comprehensive overview of the intellectual structure, collaboration patterns, or evolving thematic landscape of the field. A previous bibliometric study focusing on early mobilization analyzed research trends in ICU early activity from 2000 to 2021 and demonstrated increasing attention to its role as a potential strategy for preventing ICU-AW (11). However, because its scope was confined to early rehabilitation, it did not provide a comprehensive overview of ICU-AW–centered research across multiple disciplines or thematic domains. As research output continues to grow across multiple disciplines, a comprehensive mapping of publication trends and research networks is still lacking.

Unlike traditional narrative or systematic reviews that synthesize clinical evidence on specific questions, bibliometric analysis enables quantitative mapping of publication output, author collaborations, institutional networks, and keyword co-occurrence patterns (12). This approach facilitates a comprehensive understanding of the scope and structure of a research field, allows the tracking of emerging trends, helps identify gaps in knowledge, and informs future scientific investigations (12). Accordingly, this study employed bibliometric methods to quantitatively and qualitatively analyze publications related to ICU-AW, with the aim of evaluating their temporal and spatial distribution, research hotspots, and development trends in this field. The findings are intended to inform clinicians, rehabilitation specialists, nursing professionals, researchers, and policymakers by providing a structured overview of current research directions and potential areas for future investigation.

## Materials and methods

### Search strategy

To comprehensively search for and retrieve relevant literature in the field, various combinations of search terms related to ICU-AW and its three subtypes were used to construct the search strategy. Additionally, considering that Guillain-Barré syndrome or myasthenia gravis are common neuromuscular diseases in the ICU and are usually excluded from ICU-AW-related studies, and given that using neuromuscular disease as a search term might include more publications related to these two diseases, we excluded them during the search to avoid irrelevant retrieval. The final search strategy was as follows: (a) ICU-AW; (b) (ICU OR intensive care unit* OR critical illness*) AND (acquired weakness OR myopathy OR polyneuropathy OR polyneuromyopathy); (c) Guillain-Barre OR myasthenia gravis; (d) a OR b NOT c.

The search was conducted on August 20, 2025, the search terms were limited to topic or title/abstract, and the language was restricted to English. Given that bibliometric analyses rely heavily on citation data, the Web of Science Core Collection (WoSCC) was selected as the data source for preliminary analysis. To ensure the specificity and relevance of the retrieved records, the Web of Science Categories filter was restricted to categories most relevant to ICU-AW and with the largest number of publications: “Critical Care Medicine,” “Medicine General Internal,” “Clinical Neurology,” “Respiratory System,” “Neuroscience,” “Rehabilitation,” “Anesthesiology,” “Nursing,” “Nutrition Dietetics,” and “Surgery.” The Document Types filter was set to “Article” or “Review Article.” In total, 1,449 articles from WoSCC met the inclusion criteria and were included in the analysis. PubMed is one of the world’s largest biomedical databases and was selected as a supplementary source for external validation of ICU-AW research trends and cluster analysis. The search strategy and time range in PubMed were consistent with those used in WoSCC, and the filters were set to cover all types of clinical studies and reviews.

### Data analysis

Bibliographic records were retrieved from the database and exported in plain text format. VOSviewer (version 1.6.20; Leiden University, Netherlands) was used to visualize the publication landscape, citation patterns, and collaborative relationships within the field (13). Co-authorship, country co-occurrence, and journal citation networks were constructed to illustrate the intensity of collaboration, geographical distribution of research output, and interrelationships among leading journals. Core authors were determined based on Price’s law to ensure the inclusion of the most representative contributors (14). Considering that journals and countries with a very small number of publications may have limited representativeness in bibliometric analyses, a minimum threshold of 5 publications was applied when generating visualization maps in VOSviewer to enhance clarity and interpretability. For the analysis of author collaboration networks, only core authors within the field were included in the visualization. The weights of nodes were set to the number of documents during the analysis. In addition, CiteSpace (version 6.4. R2 Advanced; Drexel University, United States) was employed to examine the thematic evolution and emerging trends in the literature (15). A 4-year time slicing strategy was applied to balance temporal resolution and network stability, with keywords selected as the node type and the top 25 most frequent items (k = 25) extracted per slice for network construction. Cluster analysis was conducted to identify the main thematic structures of the field. Clusters were labeled using the log-likelihood ratio algorithm, and those with high silhouette values were retained. To enhance interpretative transparency, three researchers independently assessed the correspondence between each label and its constituent terms. Labels judged inappropriate by at least two reviewers were revised through discussion. High centrality and frequency terms within each cluster were preferentially considered as replacement labels. In cases where consensus was not achieved, labels were reformulated based on the shared thematic content of the cluster terms. To assess clustering quality, standard bibliometric network metrics were calculated in CiteSpace, including modularity (Q value), mean silhouette score, betweenness centrality, and overall network density. Keyword burst analysis was used to detect terms showing a rapid increase in citation or usage frequency over specific time intervals, indicating emerging research hotspots and shifts in scholarly focus. Timeline analysis was used to illustrate the chronological development of thematic clusters and the dynamic progression of research topics over time. In the visualization maps generated by VOSviewer and CiteSpace, node colors ranged from purple (earlier years) to red (more recent years), indicating the temporal distribution of nodes. To further examine the thematic stability of the findings across databases, a cross-database validation was conducted using PubMed as an independent data source. Bibliographic records were retrieved using the same search strategy and time span as the primary dataset. Given that PubMed does not provide structured citation data comparable to WoSCC, validation was limited to keyword co-occurrence clustering, burst detection, and publication trend analysis. Specifically, clustering structures were compared based on the semantic overlap of cluster labels and dominant keywords. Burst terms were examined for conceptual convergence and temporal alignment across datasets. In addition, overall publication trends were visually compared to assess consistency in temporal evolution.

### Ethical considerations

Considering that this study involved secondary analysis of previously published research articles, ethical approval was not required.

## Results

### Basic quantitative information

**Figure 1:**
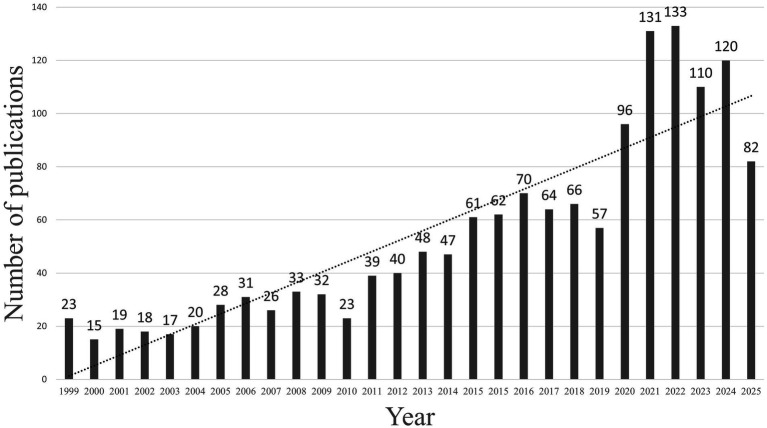
Annual number of publications on ICU-AW.

A total of 1,449 publications on ICU-AW were identified from the WoSCC, authored by 6,908 researchers affiliated with 2,209 institutions across 72 countries. These studies were published in 365 journals and collectively cited 33,490 references from 5,703 journals. As presented in Figure 1, the annual number of publications in this field has increased steadily, expanding approximately fivefold over the past two decades.

### Most productive authors

Based on Price’s law, 86 core authors were identified, each having published at least six articles. Collectively, these authors contributed 865 publications, representing 59.7% of the total publications. The 10 most productive authors are listed in Table 1. Among them, Prof. Dr. Greet Van den Berghe stands out as both the most prolific author (33 publications) and the most frequently cited (11,152 citations), and was among the earliest researchers to explore this field. The collaborative networks and temporal trends of the core authors are presented in Supplementary Figure S1, and the complete author list is provided in Supplementary Table S1.

**Table 1:** Top 10 most productive authors in ICU-AW research.

Authors	Documents	Citations	Average citation
Van Den Berghe, Greet	33	11,152	337.9
Needham, Dale M.	28	2,845	101.6
Weber-Carstens, Steffen	27	1,449	53.7
Hermans, Greet	21	3,573	170.1
Latronico, Nicola	21	2,268	108.0
Wollersheim, Tobias	17	681	40.1
Z’graggen, Werner J.	17	512	30.1
Hough, Catherine L.	16	1,671	104.4
Morris, Peter E.	14	1,164	83.1
Gosselink, Rik	13	2,287	175.9

### Most productive countries/regions

**Figure 2:**
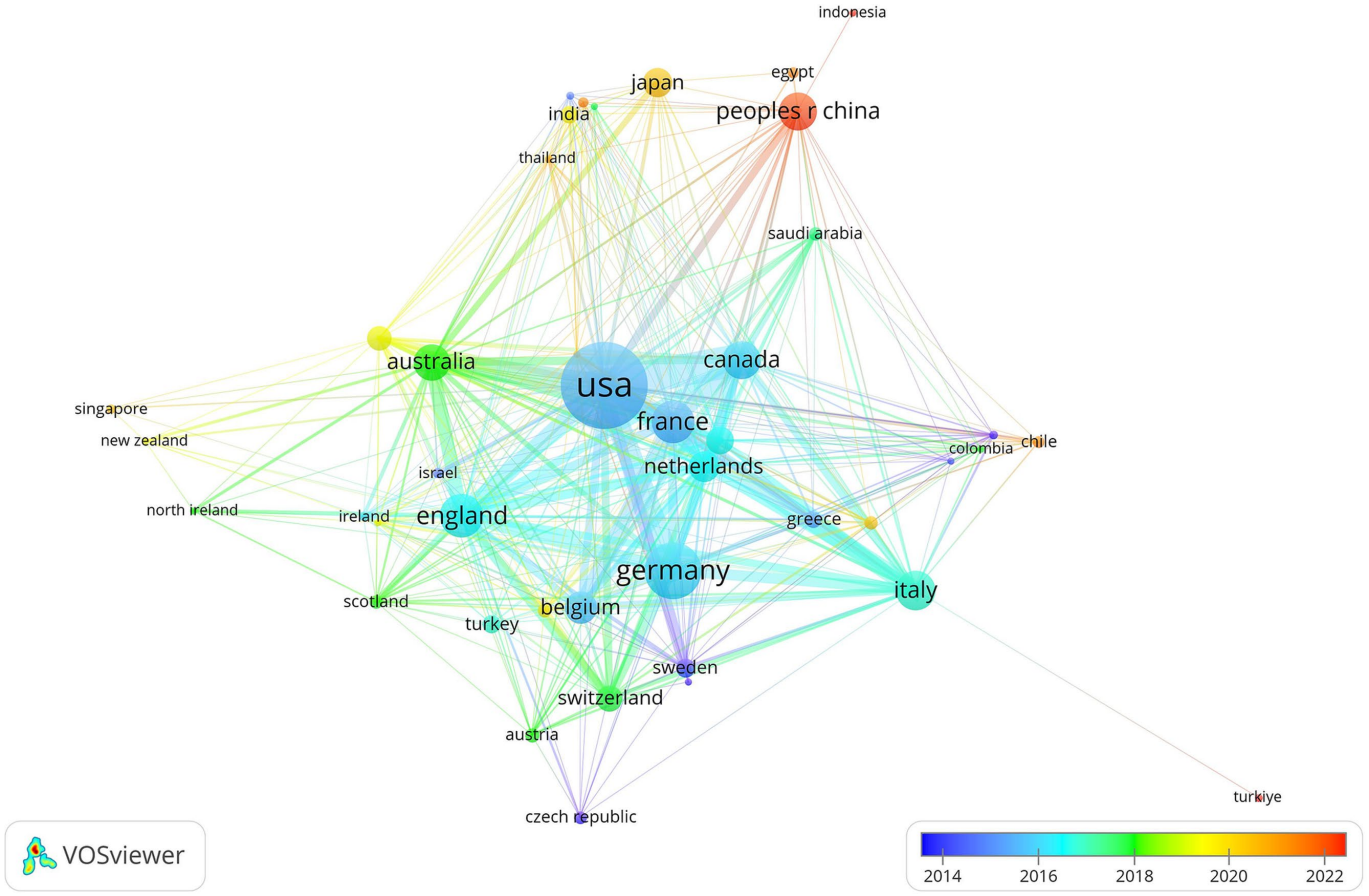
Collaboration networks and temporal distribution of major countries.

Authors from 72 countries and regions contributed to research in this field. As summarized in Table 2, the United States, Germany, and the United Kingdom were the leading contributors, with 392, 194, and 121 publications, respectively. Notably, the United States also produced the highest number of citations, indicating its dominant influence in the field. Publications from China, Japan, and Thailand were predominantly concentrated in recent years. The pattern of international collaboration and temporal distribution among these countries are illustrated in Figure 2.

**Table 2:** Top 10 contributing countries/regions in ICU-AW research.

Countries	Documents	Citations	Average citations
USA	392	22,042	56.2
Germany	194	7,851	40.5
England	121	7,345	60.7
France	120	10,133	84.4
Italy	104	4,464	42.9
Canada	97	10,204	105.2
China	97	1,976	20.4
Australia	91	4,044	44.4
Belgium	75	14,578	194.4
Netherlands	68	3,690	54.3

### Core journals

**Figure 3:**
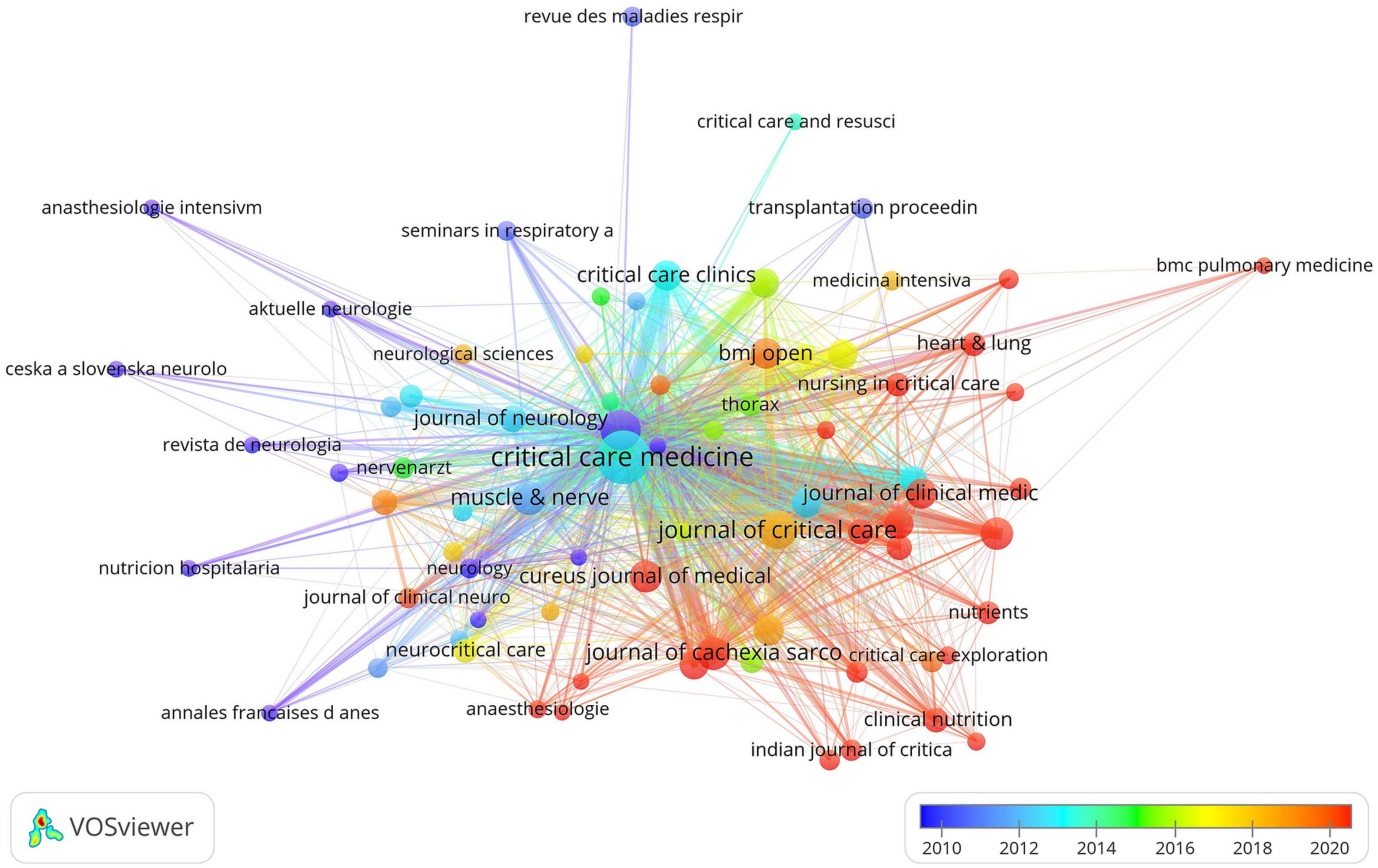
Citation relationships and temporal distribution of major journals in ICU-AW research.

A total of 365 journals have published articles in this field. Among them, Critical Care Medicine (80 publications), Intensive Care Medicine (40 publications), and Journal of Critical Care (37 publications) were the most productive outlets. Critical Care Medicine was also the most frequently cited journal, whereas the American Journal of Respiratory and Critical Care Medicine had the highest average number of citations per article. Publications in the Journal of Intensive Care Medicine, Australian Critical Care, Journal of Cachexia, Sarcopenia and Muscle, Cureus Journal of Medical Science, Clinical Nutrition, and Nutrients, etc. were largely concentrated in the later years of the study period. The top 10 journals by publication volume are listed in Table 3, while Figure 3 illustrates the citation relationships and temporal trends among the leading journals.

**Table 3:** Top 10 journals publishing ICU-AW research.

Journals	Documents	Citations	Average citations
Critical Care Medicine	80	8,984	112.3
Intensive Care Medicine	40	4,227	105.7
Journal of Critical Care	37	1,463	39.5
Muscle & Nerve	27	1,341	49.7
Journal of Cachexia Sarcopenia and Muscle	25	1,181	47.2
Current Opinion in Critical Care	24	958	39.9
Australian Critical Care	23	298	13.0
Cureus Journal of Medical Science	23	83	3.6
BMJ Open	21	418	19.9
Annals of Intensive Care	20	662	33.1

### Keyword clustering and timeline analysis

**Figure 4:**
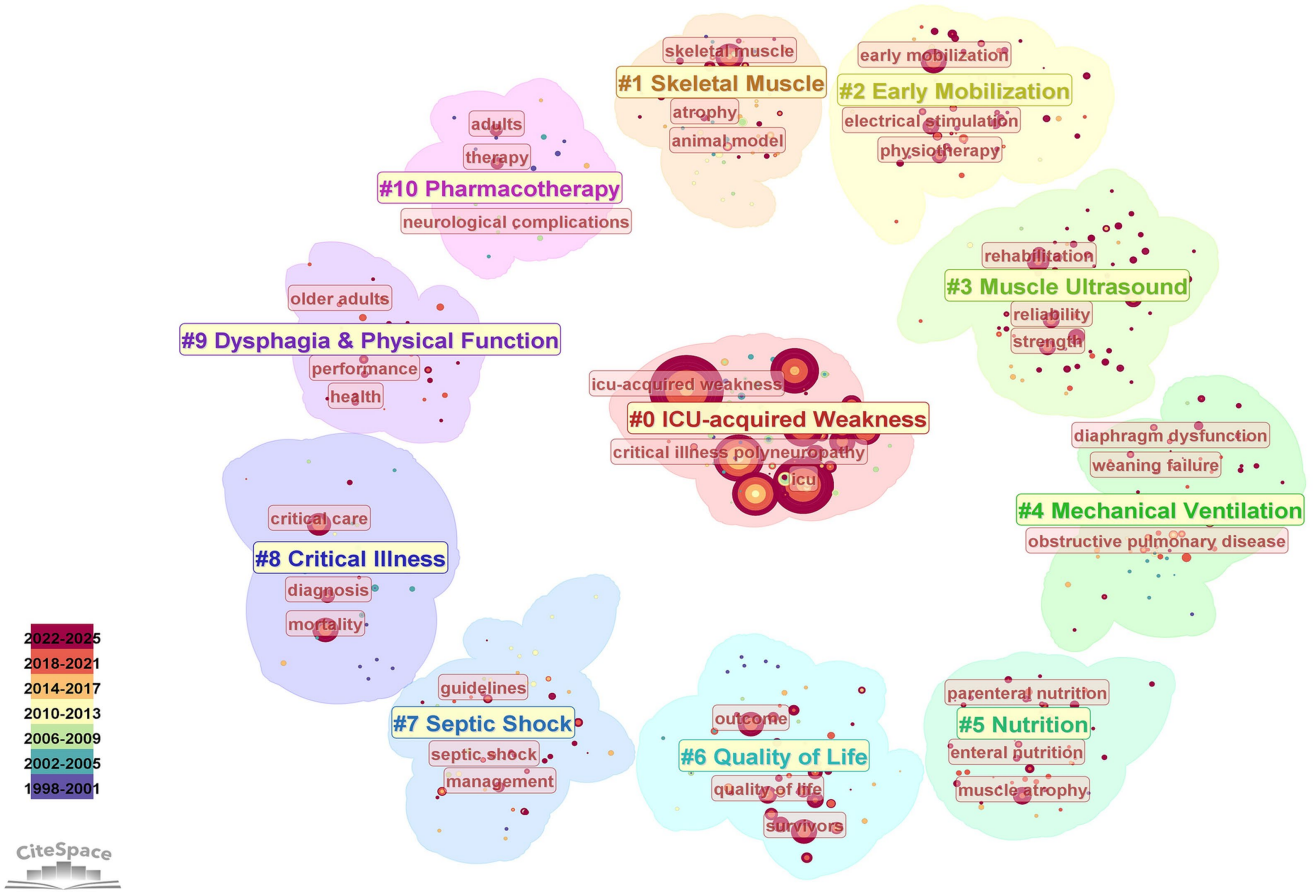
Keyword co-occurrence clusters in ICU-AW research.

The generated network consisted of 579 nodes and 2,818 links, with an overall density of 0.017, indicating a relatively sparse but structured knowledge network. The modularity value was 0.482, suggesting a significant clustering structure within the network. The mean silhouette score was 0.761, reflecting satisfactory internal consistency and reliability of the identified clusters. Nodes with high betweenness centrality include skeletal muscle (0.07), ICU-acquired weakness (0.06), outcome (0.06), critical care (0.06). Keyword clustering analysis identified 11 major clusters. As shown in Figure 4 and Supplementary Table S4, the representative clusters and their most frequently keywords include: ICU-acquired Weakness (ICU, critical illness polyneuropathy), Skeletal Muscle (animal model, atrophy), Early Mobilization (electrical stimulation, physiotherapy), Ultrasound (rehabilitation, strength, reliability), Mechanical Ventilation (obstructive pulmonary disease, weaning failure, diaphragm dysfunction), Nutrition (enteral nutrition, parenteral nutrition, muscle atrophy), Quality of Life (survivors, outcome, quality of life), Septic Shock (guidelines, management), Critical Illness (critical care, mortality, diagnosis), Dysphagia and Physical Function (older adults, performance, health), Pharmacotherapy (neurological complications, adults).

As presented in Supplementary Figure S2, clusters related to ICU-AW and skeletal muscle emerged around the year 2000 and have remained central to research over the past 25 years. Topics on early mobilization and ultrasound clusters gained prominence after 2010, while those on physical performance and nutrition clusters appeared more frequently after 2015. In contrast, keywords related to therapy have gradually declined since 2015.

### Burst keyword analysis

Burst detection identified 67 keywords with strong citation bursts between 1998 and 2025. Over the past decade, the representative burst keywords included early rehabilitation, prevention, post-intensive care syndrome, ultrasound, outcome, and diaphragmatic dysfunction (Table 4). The complete list of burst keywords is provided in Supplementary Table S2.

**Table 4:** Representative burst keywords in ICU-AW research during the past decade.

Burst keywords	Strength	Began	End
Rehabilitation	7.05	2018	2025
Outcome	5.56	2018	2021
Post-Intensive Care Syndrome	5.38	2018	2025
Ultrasound	5.19	2018	2025
Recovery	5.02	2022	2025
Covid 19	4.92	2020	2025
Limb muscle	4.86	2020	2021
Care	4.25	2018	2025
Point prevalence	4.11	2015	2021
Diaphragm dysfunction	4.1	2015	2021
Epidemiology	3.92	2022	2025
Mobilization	3.92	2017	2025
Prevention	3.71	2020	2025
Controlled mechanical ventilation	3.71	2016	2021
Early rehabilitation	3.67	2022	2025

### External validation in PubMed

A total of 648 publications were identified from PubMed database, with the annual output of ICU-AW–related studies increasing from 11 in 1999 to 86 in 2025 (Supplementary Figure S3). The clustering analysis generated a network comprising 266 nodes and 709 links, with a modularity of 0.512 and a mean silhouette score of 0.811. Seven clusters were yielded, corresponding to Critical illness myopathy, Critical Care, Mechanical Ventilation, Physical Function, Critical Illness, Neuromuscular Electrical Stimulation, and Muscle Strength (Supplementary Figure S2). The burst-term analysis demonstrated a concentrated emergence of relevant terms around 2014, primarily including “muscle atrophy,” “neuromuscular electrical stimulation,” and “neuromuscular dysfunction.” The complete list of burst terms is provided in Supplementary Table S3. No new clusters or burst terms emerged, indicating that the results remained stable and consistent.

## Discussion

### Summary

This study employed bibliometric techniques to examine research trends and hotspots in the field of ICU-AW research. The findings revealed a steady increase in publication output and identified 86 core authors who contributed 59.7% of the total publications, suggesting that the field is progressing toward a stage of relative maturity. In terms of geographical distribution, emerging research activity has been observed in Asia, particularly in East and Southeast Asia, reflecting the rapid expansion of ICU-AW studies in these regions. Citation analysis further underscored the central role of critical care journals, such as Critical Care Medicine and Intensive Care Medicine, in disseminating research findings. Meanwhile, nutrition- and neuromuscular-focused journals, including Journal of Cachexia Sarcopenia and Muscle, Muscle & Nerve and Clinical Nutrition. have increasingly appeared in recent citation networks, suggesting that ICU-AW is increasingly recognized as a multidisciplinary complication of broad clinical relevance.

Furthermore, cluster analysis identified 11 major research themes encompassing multiple disciplines, including critical care, nutrition, rehabilitation, and respiratory therapy. These clusters addressed diverse aspects of ICU-AW, including diagnosis, prevention, treatment, and prognosis, thereby reflecting the expanding scope of the field. Time-series analysis revealed heterogeneity in the evolution of these themes, demonstrating a clear shift from earlier research focused on risk factors and pharmacological interventions toward increasing emphasis on early identification, functional rehabilitation, and nutritional support. Consistent with this, keyword burst analysis supported these temporal patterns, with keywords such as ultrasound, early rehabilitation, and prevention emerging as dominant terms over the past decade. Collectively, these findings provide a comprehensive overview of the thematic evolution of ICU-AW research. They not only highlight the current hotspots and knowledge gaps, but also offer valuable insights to guide future investigations in this field.

### The trend toward non-invasive and easily accessible methods for ICU-AW identification

Since the introduction of the ICU-AW concept, its diagnosis has primarily relied on bedside manual muscle testing to assess limb strength, with the Medical Research Council (MRC) score being the most widely used tool (3). Although the MRC scale is non-invasive and inexpensive, it has several limitations, particularly its inapplicability in patients with cognitive impairment and its inability to distinguish between myopathy and neuropathy (3, 16). In early studies, muscle biopsy was employed to differentiate myopathy from neuropathy (17); however, its invasive nature and high cost restricted its application. Electrophysiological tests, while considered complementary to muscle biopsy, also require specialized expertise and equipment. Furthermore, they can be influenced by electrical interference or tissue edema, which limits its practicality in the ICU setting (18). Moreover, given the lack of specific therapeutic interventions for myopathy and polyneuropathy, establishing a definitive physiological diagnosis has not led to efficient or targeted treatment strategies (19). Therefore, over the past few decades, research efforts have gradually shifted from high-cost and less accessible methods aimed at precise pathological diagnosis to more affordable and widely available approaches focused on the identification of clinical manifestations.

Our bibliometric cluster analysis revealed the formation of the “skeletal muscle” cluster, which encompasses the keyword “atrophy,” indicating that muscle atrophy is one of the most concerning symptoms associated with ICU-AW. Furthermore, the results of timeline analysis revealed that research interest in the “muscle ultrasound” cluster began to increase around 2010–2015. The keyword “ultrasound” showed an occurrence burst in 2018, followed by “limb muscle” in 2020. These findings suggested increasing research attention to muscle ultrasonography. Ultrasound imaging of the rectus femoris muscle is commonly used to diagnose and predict ICU-AW. Although universally accepted assessment guidelines or cutoff values remain unavailable, indicators such as cross-sectional area and pennation angle have demonstrated efficacy in the diagnosis and prediction of ICU-AW (20–22). In addition, ultrasonography enables measurement of the nerve cross-sectional area, making it a potentially valuable method for differentiating myopathy from neuropathy (23).

Moreover, the frequent co-occurrence in the terms “strength” and “rehabilitation” of “muscle ultrasound” cluster suggests a growing research interest in the repeated use of ultrasound to assess muscle parameters during the recovery phase of ICU-AW. Ultrasonography can be used to evaluate the muscles, tendons, joints, soft tissues, and nerves, thereby providing valuable guidance for the development of rehabilitation plans (24). Previous studies have also demonstrated that muscle ultrasound imaging serves as an effective indicator of rehabilitation outcomes in critically ill patients (25). As an objective and highly repeatable assessment tool, ultrasound imaging is poised to play an important role in the assessment, planning, and evaluation of the rehabilitation interventions for ICU-AW in the future.

### Early rehabilitation and nutritional support as core components of ICU-AW management

The development of ICU-AW is considered to be associated with the use of multiple medications, including steroids, muscle relaxants, aminoglycoside drugs and statins, etc. (26). Therefore, reducing the use of these medications is a potential strategy for preventing ICU-AW. Medications, including growth hormone (27), beta-adrenergic agonists (28), and immunoglobulins (29) have also been reported to alleviate symptoms associated with ICU-AW. However, in the ICU setting, the primary therapeutic objective remains the management of the underlying illness rather than the direct treatment of ICU-AW.

Cluster analysis identified entries including “therapy,” “adults,” and “neurological complications” reflecting the research theme of “pharmacotherapy” in ICU-AW as a neurological complication. Notably, the results of timeline analysis show that no new entries have appeared in this theme since 2015, suggesting a decline in research activities in this area. Among pharmacological interventions, “Insulin therapy” has emerged as a recurring topic since 2005 and continues to appear in recent studies, suggesting the continued interest of researchers in this preventive strategy for ICU-AW. Previous studies have demonstrated that active blood glucose control through intensive insulin therapy can effectively reduce the risk of ICU-AW (30). Recent evidence indicates that hyperglycemia contributes to ICU-AW development through multiple interrelated mechanisms, including oxidative stress–induced mitochondrial dysfunction, activation of proteolytic and inflammatory pathways, impaired autophagy, and reduced insulin signaling–mediated protein synthesis (31).

In recent years, increasing emphasis has been placed on the importance of early rehabilitation in ICU patients. The theme “early mobilization” showed high research activity around 2010, accompanied by frequent occurrences of related terms, including “exercise rehabilitation,” “physiotherapy,” and “electrical stimulation,” as well as burst terms including “early rehabilitation” and “rehabilitation.” Furthermore, nutrition and early mobilization have recently emerged as major research hotspots, forming a distinct “nutrition” theme.

A recent review highlighted the essential role of nutritional support in critically ill patients and confirmed that adequate protein and caloric intake, combined with physical exercise, can mitigate ICU-AW and prevent muscle wasting (32). Muscle mass, a crucial indicator closely associated with both malnutrition and ICU-AW, has gained increasing attention in recent years. Although the term “muscle atrophy” first appeared in 2005 under the nutrition-related theme, its frequency has increased rapidly since 2022. The pathophysiological process of ICU-AW primarily involves muscle catabolism and dysfunction caused by inflammation, oxidative stress, mitochondrial impairment, and prolonged immobilization (33). Nutritional support provides essential substrates for muscle repair and protein synthesis, whereas early mobilization promotes muscle regeneration and functional recovery. Collectively, these patterns indicate that multidimensional interventions combining early mobilization and nutritional support have become a prominent research focus.

### From survival to patient-centered outcomes: changing ICU-AW endpoints

With the substantial decline in ICU mortality in recent years, an increasing number of patients now survive critical illnesses and are discharged from the ICU (34). However, surviving a critical illness does not mean achieving full recovery or long-term health (35). Previous studies have shown that within 1 year after ICU discharge, 58% of medical patients, 64% of emergency surgical patients, and 43% of elective surgical patients experience physical, psychological, or cognitive impairments (36). Consequently, research attention has increasingly shifted toward patient-centered outcomes such as physical function and quality of life, which are essential for survivors’ reintegration into their family and professional roles. In our cluster analysis, the emergence of clusters such as “quality of life” and “dysphagia and physical function” reflects this evolving focus in ICU-AW research. Health-related quality of life (HRQoL) is a multidimensional outcome encompassing physical, psychological, cognitive, and social domains, representing the extent to which a patient’s life is affected by illness or medical care (37). A previous meta-analysis demonstrated that ICU-AW has a detrimental impact on patients’ quality of life over the long term (38), highlighting the growing research attention to post-ICU outcomes identified in our bibliometric analysis.

In recent years, dysphagia resulting from ICU-AW-related neuromuscular dysfunction has gained increasing recognition (39). Among patients in the ICU, swallowing disorders can increase the risk of pneumonia, prolonged ICU length of stay, increased healthcare costs, and higher mortality rates (40). In ICU-AW research, dysphagia has been identified both as an adverse outcome (40) and as a significant risk factor for malnutrition (41). Malnutrition, in turn, may further impair muscle structure and physical function, thereby exacerbating ICU-AW symptoms. The results of our cluster analysis showed that dysphagia clustered with physical performance, whereas nutrition clustered with muscle atrophy. The inclusion of structure- and function-related terms such as “performance” and “muscle atrophy” within these clusters reflects increasing research attention to the relationship between swallowing impairment, muscle wasting, and functional decline in ICU-AW. Furthermore, the results of the burst keyword analysis identified the emergence of the term “post-intensive care syndrome” (PICS), indicating increasing research attention to long-term physical, psychological, and cognitive outcomes after critical illness. As ICU-AW is recognized as a component of PICS, the appearance of this term reflects a broader shift in research focus from acute survival to long-term functional and quality-of-life outcomes. In summary, the focus of ICU-AW research has gradually shifted from concerns about mortality and acute complications toward quality of life and holistic health.

### Strengths and limitations

This study provides a comprehensive overview of more than two decades of research on ICU-AW by analyzing the geographical, temporal, and journal distributions of publications in this field. Using cluster analysis, timeline analysis, and burst keyword detection, we identified the major research themes and their evolving trends. These findings offer valuable insights into the current research landscape and highlight potential directions for future studies on ICU-AW.

However, certain limitations should be acknowledged. First, due to the inherent constraints of the selected database, only studies published after 1999 were included, which may have resulted in the omission of earlier relevant literature. In addition, the analysis was limited to English-language publications, which may have introduced language bias and led to the underrepresentation of studies published in other languages. Second, citation-based indicators are influenced by the time required for articles to accumulate citations; therefore, recently published studies may be underrepresented due to the citation lag effect. Citation counts may also be affected by self-citation practices. Third, the bibliometric approach focuses on keyword clustering and citation network analysis rather than on a systematic evaluation or quantitative synthesis of the study findings, and the results of bibliometric analyses may vary depending on the algorithms and parameter settings of the visualization software used. Therefore, it cannot provide definitive conclusions regarding the effectiveness or validity of specific interventions or mechanisms. To address clinically relevant questions more comprehensively, future research should integrate bibliometric analyses with systematic reviews and original studies. Finally, owing to the absence of structured citation data in PubMed, cross-database validation was confined to qualitative comparisons of keyword clustering and temporal patterns rather than quantitative replication of citation networks.

## Conclusion

Over the past two decades, research on ICU-AW has steadily increased, with the annual number of publications rising from approximately 15–20 to approximately 120–130. This bibliometric analysis provides a comprehensive overview of the global research landscape in this field. The findings indicate that research activity has been predominantly led by European and North American countries, while contributions from East Asian countries have expanded considerably in recent years. Although most studies have been published in journals focusing on critical care medicine, publications in nutrition- and neuromuscular-focused journals have become increasingly common. A total of 11 major research clusters were identified, encompassing the diagnosis, prevention, treatment, and prognosis of ICU-AW. Overall, these findings map the evolution of research themes and may help identify emerging directions and generate hypotheses for future studies.
