# Supplementary material for: Mapping the research landscape and evolving hotspots of intensive care unit-acquired weakness: a dual-database bibliometric analysis
Source: Front Neurol. 2026 Apr 8;17:1759594. doi: 10.3389/fneur.2026.1759594 (PMC13099319; doi:10.3389/fneur.2026.1759594)
Supplement: Supplementary file 1 [file Data_Sheet_1.DOCX]

**Mapping the research landscape and evolving hotspots of intensive care unit–acquired weakness: A bibliometric and visualization review**

Supplementary Material

Tables

Table S1: Core authors of intensive care unit-acquired weakness research.

Table S2: Burst keywords of intensive care unit-acquired weakness research in Web of Science Core Collection.

Table S3: Burst keywords of intensive care unit-acquired weakness research in PubMed.

Table S4. Clustering labels and keywords.

Figures

Figure S1: Collaboration networks and temporal distribution of core authors.

Figure S2: Timeline analysis of major keyword clusters in ICU-AW research.

Figure S3: Keyword co-occurrence clusters in ICU-AW research in PubMed.

Table S1: Core authors of intensive care unit-acquired weakness research.

| Author | Documents | Citations | Avergae citation |
| --- | --- | --- | --- |
| Van Den Berghe, Greet | 33 | 11152 | 337.9 |
| Needham, Dale M. | 28 | 2845 | 101.6 |
| Weber-Carstens, Steffen | 27 | 1449 | 53.7 |
| Hermans, Greet | 21 | 3573 | 170.1 |
| Latronico, Nicola | 21 | 2268 | 108.0 |
| Wollersheim, Tobias | 17 | 681 | 40.1 |
| Z'graggen, Werner J. | 17 | 512 | 30.1 |
| Hough, Catherine L. | 16 | 1671 | 104.4 |
| Morris, Peter E. | 14 | 1164 | 83.1 |
| Gosselink, Rik | 13 | 2287 | 175.9 |
| Horn, Janneke | 13 | 413 | 31.8 |
| Mayer, Kirby P. | 13 | 181 | 13.9 |
| Moss, Marc | 13 | 1702 | 130.9 |
| Schaller, Stefan J. | 13 | 246 | 18.9 |
| Denehy, Linda | 12 | 1013 | 84.4 |
| Grunow, Julius J. | 12 | 236 | 19.7 |
| Hart, Nicholas | 12 | 1231 | 102.6 |
| Larsson, Lars | 12 | 468 | 39.0 |
| Mehrholz, Jan | 12 | 169 | 14.1 |
| Rich, Mark M. | 12 | 836 | 69.7 |
| Sharshar, Tarek | 12 | 1826 | 152.2 |
| Wouters, Pieter J. | 12 | 2483 | 206.9 |
| Ely, EE. Wesley | 11 | 1679 | 152.6 |
| Herridge, Margaret S. | 11 | 1449 | 131.7 |
| Parry, Selina M. | 11 | 912 | 82.9 |
| Schefold, Joerg C. | 11 | 700 | 63.6 |
| Wieske, Luuk | 11 | 379 | 34.5 |
| Bostock, Hugh | 10 | 249 | 24.9 |
| Demoule, Alexandre | 10 | 547 | 54.7 |
| Fielitz, Jens | 10 | 548 | 54.8 |
| Kress, John P. | 10 | 1761 | 176.1 |
| Pohl, Marcus | 10 | 202 | 20.2 |
| Casaer, Michael P. | 9 | 993 | 110.3 |
| Connolly, Bronwen | 9 | 406 | 45.1 |
| De Jonghe, Bernard | 9 | 1990 | 221.1 |
| Dres, Martin | 9 | 741 | 82.3 |
| Fan, Eddy | 9 | 1146 | 127.3 |
| Hall, Jesse B. | 9 | 1514 | 168.2 |
| Hodgson, Carol L. | 9 | 518 | 57.6 |
| Karatzanos, Eleftherios | 9 | 843 | 93.7 |
| Koch, Susanne | 9 | 478 | 53.1 |
| Nakamura, Kensuke | 9 | 211 | 23.4 |
| Puthucheary, Zudin | 9 | 918 | 102.0 |
| Schultz, Marcus J. | 9 | 341 | 37.9 |
| Annane, Djillali | 8 | 913 | 114.1 |
| Batt, Jane | 8 | 454 | 56.8 |
| Berney, Sue | 8 | 649 | 81.1 |
| Bruyninckx, Frans | 8 | 979 | 122.4 |
| Colantuoni, Elizabeth | 8 | 817 | 102.1 |
| Gunst, Jan | 8 | 497 | 62.1 |
| Hund, E | 8 | 414 | 51.8 |
| Nakanishi, Nobuto | 8 | 95 | 11.9 |
| Nanas, Serafim | 8 | 790 | 98.8 |
| Routsi, Christina | 8 | 723 | 90.4 |
| Spies, Claudia D. | 8 | 529 | 66.1 |
| Spranger, Joachim | 8 | 401 | 50.1 |
| Spuler, Simone | 8 | 622 | 77.8 |
| Tankisi, Hatice | 8 | 156 | 19.5 |
| Van Schaik, Ivo N. | 8 | 334 | 41.8 |
| Verhamme, Camiel | 8 | 334 | 41.8 |
| Witteveen, Esther | 8 | 216 | 27.0 |
| Blobner, Manfred | 7 | 274 | 39.1 |
| Derde, Sarah | 7 | 571 | 81.6 |
| Ehler, Johannes | 7 | 56 | 8.0 |
| Eikermann, Matthias | 7 | 237 | 33.9 |
| Gerovasili, Vasiliki | 7 | 692 | 98.9 |
| Gonzalez-Seguel, Felipe | 7 | 41 | 5.9 |
| Jaber, Samir | 7 | 2076 | 296.6 |
| Papazian, Laurent | 7 | 1918 | 274.0 |
| Preiser, Jean-Charles | 7 | 400 | 57.1 |
| Van Der Schaaf, Marike | 7 | 413 | 59.0 |
| Vanhorebeek, Ilse | 7 | 916 | 130.9 |
| Carbon, Niklas M. | 6 | 104 | 17.3 |
| Dos Santos, Claudia C. | 6 | 273 | 45.5 |
| Hedstrom, Yvette | 6 | 271 | 45.2 |
| Herridge, Margaret | 6 | 355 | 59.2 |
| Heunks, Leo M. A. | 6 | 681 | 113.5 |
| Hopkins, Ramona O. | 6 | 930 | 155.0 |
| Kho, Michelle E. | 6 | 221 | 36.8 |
| Klawitter, Felix | 6 | 36 | 6.0 |
| Mendez-Tellez, Pedro A. | 6 | 1135 | 189.2 |
| Similowski, Thomas | 6 | 541 | 90.2 |
| Sonoo, Tomohiro | 6 | 203 | 33.8 |
| Supinski, Gerald S. | 6 | 180 | 30.0 |
| Weijs, Peter J. M. | 6 | 501 | 83.5 |
| Wilmer, Alexander | 6 | 1060 | 176.7 |

Table S2: Burst keywords of intensive care unit-acquired weakness research in Web of Science Core Collection.

| Keywords | Year | Strength | Begin | End |
| --- | --- | --- | --- | --- |
| critical illness polyneuropathy | 1999 | 25.02 | 1999 | 2017 |
| multiple organ failure | 1999 | 24.85 | 1999 | 2013 |
| sepsis | 1999 | 22.2 | 1999 | 2013 |
| failure | 1999 | 5.8 | 1999 | 2013 |
| neurological complications | 1999 | 4.2 | 1999 | 2013 |
| critical illness myopathy | 1999 | 25.12 | 1999 | 2009 |
| ill patients | 1999 | 18.64 | 1999 | 2009 |
| prolonged paralysis | 1999 | 10.68 | 1999 | 2009 |
| neuromuscular blockade | 1999 | 10.03 | 1999 | 2009 |
| status asthmaticus | 1999 | 8.34 | 1999 | 2009 |
| complications | 1999 | 6.24 | 1999 | 2009 |
| intensive care | 1999 | 8.7 | 1999 | 2005 |
| necrotizing myopathy | 1999 | 3.97 | 1999 | 2001 |
| direct muscle stimulation | 2000 | 4.88 | 2000 | 2017 |
| blocking agents | 2000 | 9.27 | 2000 | 2013 |
| organ failure | 2000 | 6.25 | 2000 | 2013 |
| neuromuscular dysfunction | 2001 | 3.46 | 2001 | 2013 |
| tumor necrosis factor | 2001 | 3.42 | 2001 | 2009 |
| inflammatory response syndrome | 2002 | 9.21 | 2002 | 2017 |
| septic shock | 2002 | 5.31 | 2002 | 2013 |
| muscle | 2002 | 6.65 | 2002 | 2009 |
| myosin | 2002 | 5.84 | 2002 | 2009 |
| corticosteroids | 2002 | 5.53 | 2002 | 2009 |
| sofa score | 2002 | 3.45 | 2002 | 2009 |
| animal model | 2003 | 7.04 | 2003 | 2009 |
| intensive insulin therapy | 2004 | 10.65 | 2004 | 2013 |
| fast inactivation | 2005 | 6.49 | 2005 | 2013 |
| rat model | 2005 | 5.45 | 2005 | 2013 |
| peripheral nerve | 2006 | 5.75 | 2006 | 2017 |
| care unit | 2006 | 3.47 | 2006 | 2017 |
| obstructive pulmonary disease | 2002 | 3.42 | 2006 | 2017 |
| risk factors | 2003 | 13.14 | 2006 | 2013 |
| insulin therapy | 2006 | 6.53 | 2006 | 2013 |
| acute respiratory distress syndrome | 2002 | 6.53 | 2006 | 2013 |
| neuromuscular manifestations | 2006 | 4.49 | 2006 | 2013 |
| critical illness polyneuromyopathy | 1999 | 3.98 | 2006 | 2013 |
| oxidative stress | 2009 | 4.3 | 2009 | 2017 |
| severe sepsis | 2006 | 5.23 | 2010 | 2021 |
| randomized controlled trial | 2010 | 6.61 | 2010 | 2017 |
| acute lung injury | 2003 | 5.55 | 2010 | 2017 |
| acquired paresis | 2010 | 4.73 | 2010 | 2017 |
| confusion assessment method | 2010 | 4.13 | 2010 | 2017 |
| quality improvement | 2010 | 3.94 | 2010 | 2017 |
| respiratory failure | 1999 | 3.38 | 2010 | 2013 |
| long term outcm | 2012 | 4.9 | 2012 | 2017 |
| interobserver agreement | 2012 | 4.64 | 2012 | 2017 |
| human skeletal muscle | 2012 | 3.49 | 2012 | 2017 |
| critically ill | 2014 | 5.33 | 2014 | 2021 |
| length of stay | 2014 | 4.16 | 2014 | 2021 |
| strength | 2009 | 3.85 | 2014 | 2021 |
| phrenic nerve stimulation | 2002 | 3.58 | 2014 | 2021 |
| critically ill patients | 1999 | 4.43 | 2014 | 2017 |
| point prevalence | 2015 | 4.11 | 2015 | 2021 |
| diaphragm dysfunction | 2015 | 4.1 | 2015 | 2021 |
| controlled mechanical ventilation | 2016 | 3.71 | 2016 | 2021 |
| mobilization | 2017 | 3.92 | 2017 | 2025 |
| rehabilitation | 2012 | 7.05 | 2018 | 2025 |
| post-intensive care syndrome | 2018 | 5.38 | 2018 | 2025 |
| ultrasound | 2015 | 5.19 | 2018 | 2025 |
| care | 2006 | 4.25 | 2018 | 2025 |
| outcome | 2000 | 5.56 | 2018 | 2021 |
| covid 19 | 2020 | 4.92 | 2020 | 2025 |
| prevention | 2020 | 3.71 | 2020 | 2025 |
| limb muscle | 2020 | 4.86 | 2020 | 2021 |
| recovery | 2014 | 5.02 | 2022 | 2025 |
| epidemiology | 2016 | 3.92 | 2022 | 2025 |
| early rehabilitation | 2014 | 3.67 | 2022 | 2025 |

Table S3. Burst keywords of intensive care unit-acquired weakness research in PubMed.

| Keywords | Year | Strength | Begin | End |
| --- | --- | --- | --- | --- |
| intensive care unit | 2014 | 3.43 | 2022 | 2025 |
| muscle atrophy | 2017 | 1.96 | 2020 | 2025 |
| intensive care unit-acquired weakness | 2014 | 1.83 | 2014 | 2017 |
| muscle wasting | 2015 | 1.76 | 2022 | 2023 |
| neuromuscular dysfunction | 2018 | 1.58 | 2018 | 2019 |
| neuromuscular electrical stimulation | 2017 | 1.57 | 2017 | 2023 |
| critically ill | 2017 | 1.23 | 2017 | 2019 |
| intensive care | 2014 | 1.2 | 2014 | 2017 |
| post-icu syndrome | 2016 | 1.17 | 2016 | 2017 |
| bed rest | 2015 | 1.16 | 2015 | 2017 |
| critically ill patients | 2019 | 1.15 | 2019 | 2023 |
| electrical stimulation | 2014 | 1.12 | 2020 | 2023 |
| intensive care unit patients | 2020 | 1.09 | 2020 | 2021 |
| older adults | 2020 | 1.09 | 2020 | 2021 |
| central nervous system | 2020 | 1.09 | 2020 | 2021 |
| chronic critical illness | 2021 | 1.07 | 2021 | 2025 |
| subarachnoid hemorrhage | 2022 | 1.07 | 2022 | 2023 |
| functional status | 2023 | 1.07 | 2023 | 2025 |
| cognitive impairment | 2019 | 1.06 | 2019 | 2023 |
| enteral nutrition | 2018 | 1.05 | 2018 | 2019 |
| muscular diseases | 2018 | 1.05 | 2018 | 2019 |
| icu-acquired muscle weakness | 2016 | 1.03 | 2016 | 2019 |
| critical illness polyneuropathy | 2013 | 1.02 | 2022 | 2023 |

Table S4. Clustering labels and keywords.

| No. | LLR algorithm generated labels | Manually changed labels | Silhouette | Keywords^*^ |
| --- | --- | --- | --- | --- |
| 0 | Critical Illness Polyneuropathy | ICU-acquired Weakness | 0.771 | icu-acquired weakness, complications, icu, neuromuscular blockade, mechanical ventilation, ill patients, disorders, neuromuscular dysfunction, critically ill patients, muscle weakness, myosin, critical illness, critical illness myopathy, risk factors, sepsis, intensive care, critical illness polyneuromyopathy, muscle, failure, blocking-agents, inflammation, nerve conduction studies, acute respiratory distress syndrome, multiple organ failure, insulin therapy, care, inflammatory response syndrome, intensive insulin therapy, blocking agents, prolonged paralysis, peripheral nerve, status asthmaticus, tumor necrosis factor, corticosteroids, neuromuscular complications, abnormality, prolonged neuromuscular blockade, diaphragmatic fatigue, critical illness polyneuropathy, recovery, direct muscle stimulation, acquired paresis, neuromuscular manifestations, necrotizing myopathy, sofa score, acute severe asthma, lung injury, muscle biopsy, severe asthma, systemic inflammatory response syndrome, vecuronium, italian multicenter crimyne, pancuronium bromide, action potential duration, agents, clinical findings, growth hormone, long term infusion, multiple organ dysfunction syndrome, acute corticosteroid myopathy, acute flaccid paralysis, autoimmune diseases, experimental rat model, hydrocortisone, infusion, muscle paralysis, muscle relaxants, organ dysfunction/failure, septic syndrome, united states, acidosis, acquired neuromuscular disorders, acute muscle disease, air flow limitation, alcohol abuse, artificial ventilation, bromide, cell culture assay, cervical magnetic stimulation |
| 1 | Skeletal Muscle | / | 0.82 | skeletal muscle, atrophy, animal model, mechanisms, models, protein synthesis, fibers, mitochondrial dysfunction, activation, gene expression, insulin, excitability, human skeletal muscle, expression, fast inactivation, inactivation, cecal ligation, action potentials, acetylcholine receptors, neuromuscular blocking agent, ubiquitin ligases, rat model, nerve conduction, efficacy, human quadriceps muscle, muscle velocity recovery cycles, sodium channel, velocity recovery cycles, chronic sepsis, degradation, endotoxin, membrane dysfunction, men, nerve excitability changes, protein breakdown, sodium channels, cachexia, calcium regulation, denervation, differentiation factor 15, electrical excitability, electromyography, extracellular matrix, fatty acid oxidation, foxo transcription factors, frequency ramp, growth, humans, increases, induced hyperkalemia, laboratory models, measurement and monitoring, mitochondrial function, molecular mechanisms, motor units, multiorgan failure, muscle contraction, muscle membrane potential, muscle velocity recovery cycle, myofibrillar protein oxidation, myosin heavy chain, na+ channels, nerve conduction study, neuromuscular transmission, protein kinase c, rat soleus, release, slow inactivation, acetylcholine receptor, adult skeletal muscle, c protein |
| 2 | Early Mobilization | / | 0.66 | acute respiratory failure, respiratory failure, bed rest, early mobilization, physiotherapy, exercise rehabilitation, sedation, physical rehabilitation, patient, mobility, neuromuscular weakness, occupational therapy, care unit, muscle stimulation, electrical stimulation, length of stay, early ambulation, ecmo, quality improvement, pediatrics, safety, distress syndrome, physical activity, quality, brain injury, traumatic brain injury, society, long term survival, care unit patients, early rehabilitation, clinical practice guidelines, point prevalence, discharge, clinical practice guideline, implementation, recommendations, stay, artificial intelligence, critically ill children, ill, length, program, virtual reality, functional recovery, interrater reliability, neurocritical care, physical therapy modalities, predicts mortality, protocol, trials, abcde bundle, actigraphy, activity patterns, clinical trials, comorbidity index, damage, drugs, early mobilization practices, ergometer training, experience, external validation, extracorporeal membrane oxygenation (ecmo), functional capacity, functional independence measure, hospital discharge, icu mobility, lung transplantation, mobilizing patients |
| 3 | Ultrasound | Muscle Ultrasound | 0.649 | validity, multicenter, strength, reliability, cross sectional area, stimulation, magnetic stimulation, diaphragm, rehabilitation, ultrasound, critically ill, paresis, thickness, mobilization, unit, feasibility, score, sarcopenia, scale, nerve, critical care outcomes, mass, impairment, quantitative neuromuscular ultrasound, abcdef bundle, anxiety, quadriceps, responsiveness, endotracheal intubation, frailty, barthel index, cycle ergometry, functional outcm, tool, cystic fibrosis, functional outcome, involvement, neurological manifestations, practice guidelines, quadriceps femoris, quadriceps muscle, quadriceps strength, reference values, skeletal, skeletal muscle function, admission, anesthesia, benzodiazepine, body composition changes, chronaxie, consensus, consequences, detecting aspiration, disuse, early rehabilitation training, gait assessment, intensive care unit-acquired paresis, knee extensor muscles, multiple organ dysfunction, muscle ultrasonography, neuromuscular ultrasound |
| 4 | Mechanical Ventilation | / | 0.766 | chronic obstructive pulmonary disease, weaning failure, obstructive pulmonary disease, oxidative stress, phrenic nerve stimulation, heart failure, spontaneous breathing trial, nitric oxide synthase, diaphragm dysfunction, epidemiology, injury, contractile property, extubation, noninvasive ventilation, pressure, case report, respiratory muscles, duration, rat diaphragm, necrosis factor alpha, paralysis, contractile dysfunction, 1 year mortality, cohort, limb muscle, controlled mechanical ventilation, randomized trial, critical care nursing, diaphragm weakness, diaphragm ultrasound, in vivo, liberation, limb muscle weakness, maximal inspiratory pressure, pressure release ventilation, pressure support ventilation, prolonged weaning, pulmonary rehabilitation, amyotrophic lateral sclerosis, blood flow, cardiac dysfunction, chest physiotherapy, classification, cough strength, diaphragm atrophy, diaphragmatic weakness, distress, disuse muscle atrophy, esophageal pressure, functional outcomes, induced diaphragmatic dysfunction, neuromuscular blockers, phrenic nerve injury, proportional assist ventilation, rat skeletal muscle, transdiaphragmatic pressure, upper abdominal surgery, airway occlusion pressure, axonal polyneuropathy |
| 5 | Nutrition | / | 0.737 | parenteral nutrition, muscle atrophy, association, body composition, metabolism, risk, enteral nutrition, muscle mass, protein, energy, trial, requirements, severity, critically ill patient, indirect calorimetry, late parenteral nutrition, muscle protein synthesis, amino acids, critical care nutrition, clinical trial, nutrition, covid 19, prevalence, immunosuppression, persistent inflammation, autophagy, area, adipose tissue, anabolic resistance, clinical practice, d deficiency, early parenteral nutrition, enteral feeding, hydroxy beta methylbutyrate, impedance analysis, index, obesity, older, sars cov 2, standard, active mobilization, beta hydroxybutyrate, bioelectrical impedance analysis, body mass index, brain, clinical outcm, cohort studies, critical patient, energy balance, generation, hospital mortality, infection, aids |
| 6 | Quality of Life | / | 0.76 | outcome, acute lung injury, posttraumatic stress disorder, delirium, survivors, quality of life, impact, interobserver agreement, cognitive impairment, prolonged mechanical ventilation, pulmonary function, end expiratory pressure, muscle strength, handgrip strength, physical function, functional status, functional disability, disability, long term outcm, support, long term, post-intensive care syndrome, randomized controlled trial, hand held dynamometry, prevention, follow up, depression, recovery of function, symptoms, central nervous system, coronavirus, exercise capacity, cardiac arrest, intracranial pressure, muscle strength assessment, cerebrospinal fluid, coronavirus infection, disease management program, exercise tolerance, health status, muscle strength dynamometer, muscular dystrophy, patterns, term follow up, 5 year, abdominal compartment syndrome, acute myositis, antimicrobial treatment, brachial plexus injury, cardiopulmonary bypass, carotid endarterectomy |
| 7 | Septic Shock | / | 0.763 | chronic critical illness, guidelines, severe sepsis, organ failure, septic shock, double blind, acute kidney injury, glucose control, confusion assessment method, cardiac surgery, community acquired pneumonia, acute respiratory distress, growth factor i, c reactive protein, emergency department, low risk patients, management, systematic review, predictors, computed tomography, brain dysfunction, creatine kinase, level, blood brain barrier, controlled trial, icu acquired paresis, lipopolysaccharide, long-term outcome, meta-analysis, morbidity, prediction model, septic encephalopathy, ventilator associated pneumonia, cam icu, care medicine sccm, central pontine myelinolysis, coronary, daily interruption, daily sedative interruption, definitions, delirium monitoring/management, dexamethasone, dexmedetomidine, european society, evidence-based medicine, inflammatory response, low dose hydrocortisone, myocardial infarction, catecholamine support |
| 8 | Critical Illness | / | 0.899 | critical care, mortality, disease, acute renal failure, stroke, acute stroke, diagnosis, rehabilitation medicine, peripheral neuropathy, acute myocardial infarction, diabetes mellitus, hyperglycemia, pediatric intensive care, adult intensive &, autonomic dysfunction, criteria, heart rate variability, interleukin 6, organ dysfunction syndrome, stress, 9 year experience, acute physiology, admission hyperglycemia, antibiotic prophylaxis, blood cultures, blood glucose level, brain swelling/edema, cerebral perfusion pressure |
| 9 | Swallowing | Dysphagia & Physical Function | 0.846 | older adults, performance, covid-19, differentiation, fiberoptic endoscopic evaluation, aspiration, population, cardiac rehabilitation, balance, inpatient rehabilitation, health, time, activities of daily living, clinical characteristics, medicine, prolonged endotracheal intubation, common, dysphagia, infections, grip, oropharyngeal dysphagia, clinically important difference, cytokine storm, deglutition disorder, disruption, dynamometry |
| 10 | Neurological Complications | Pharmacotherapy | 0.905 | therapy, neurological complications, adults, blood glucose control, pharmacokinetics, encephalopathy, crucial role, head injury, liver transplantation, spectrum, apoptosis, calcium release, continuous electroencephalography, cyclosporine, dermatomyositis, exposure, intermediate syndrome, lipid lowering drugs, mucular diseases, pancuronium, polymyositis, statin associated myopathy, steroid induced myopathy, abstinent, acute carbamate, calcitonin |

^*^Sort by centrality from highest to lowest

Abbreviation: LLR, log-likelihood ratio.


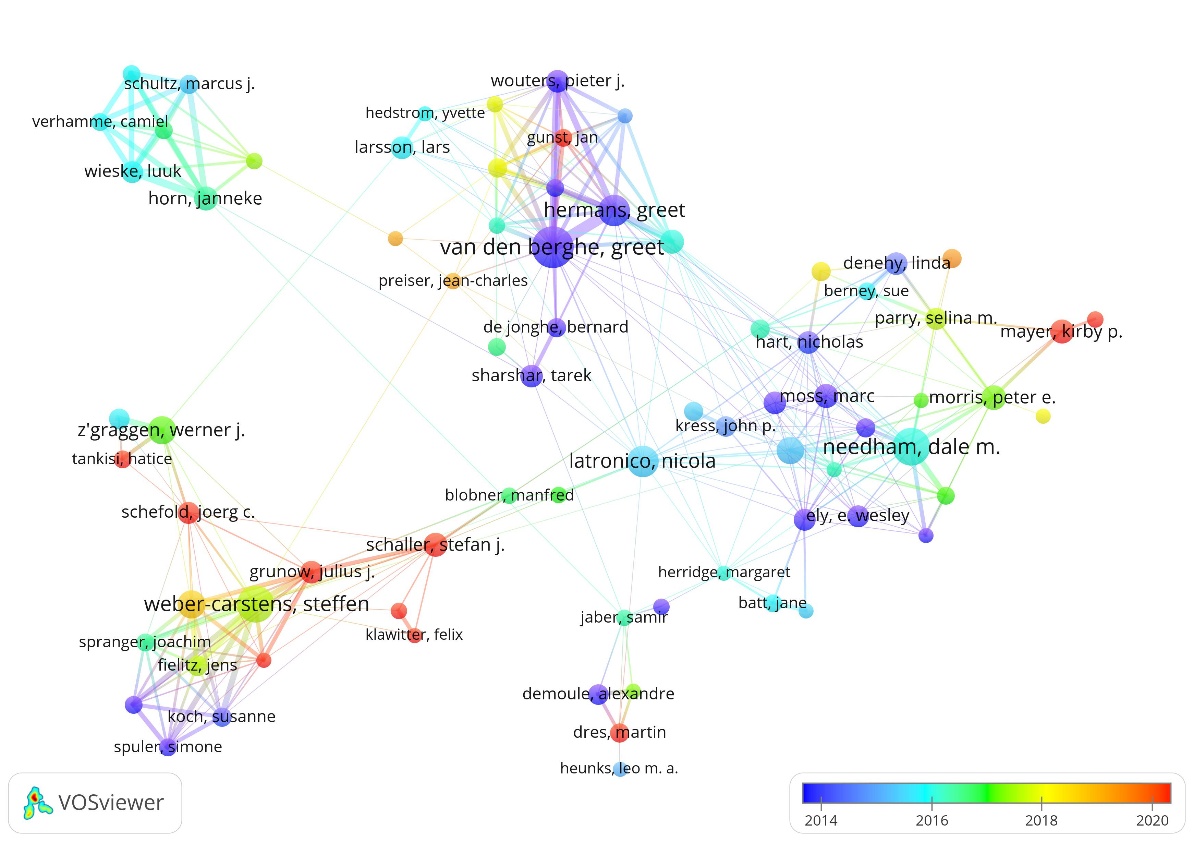


Figure S1: Collaboration networks and temporal distribution of core authors.


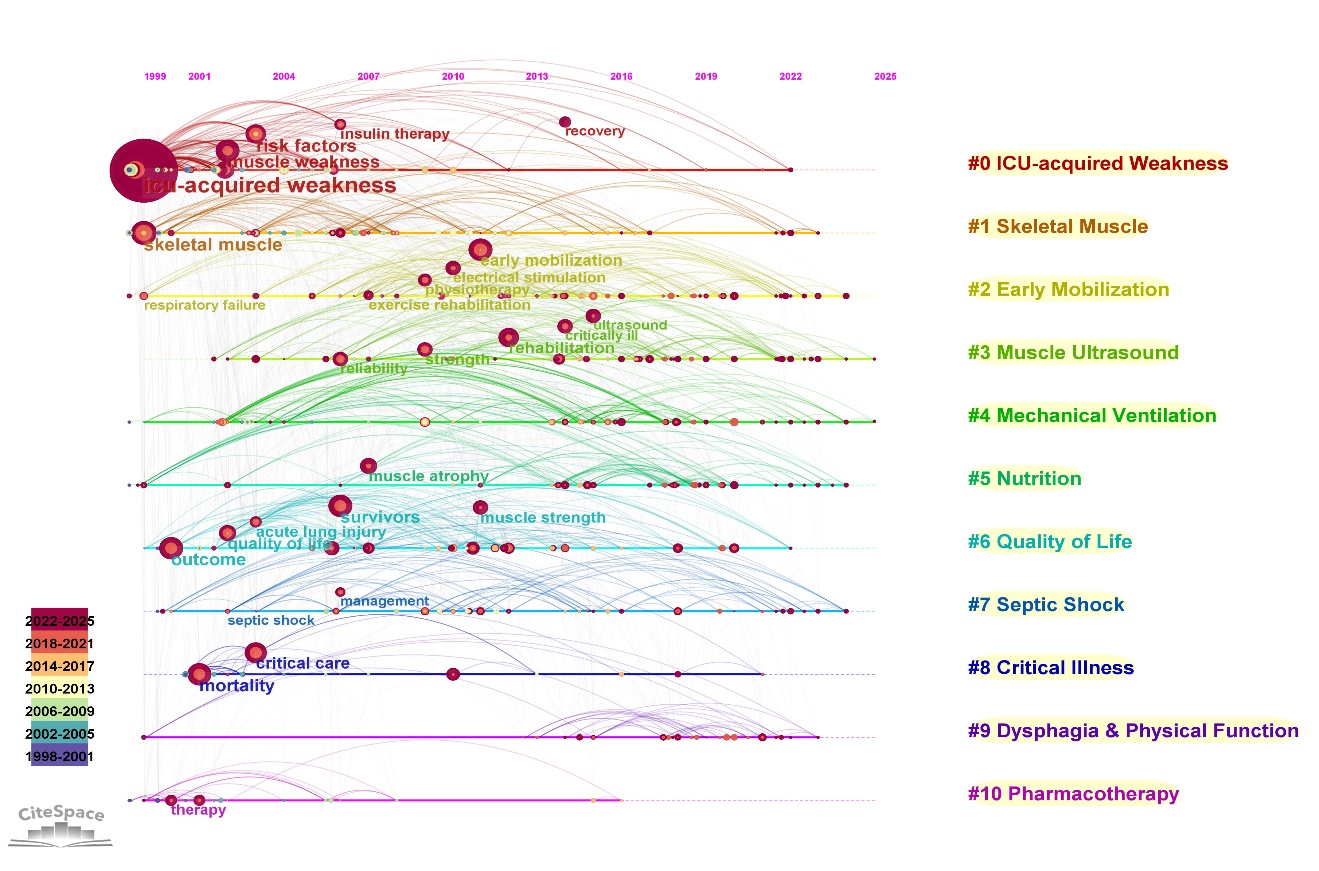


Figure S2: Timeline analysis of major keyword clusters in ICU-AW research.


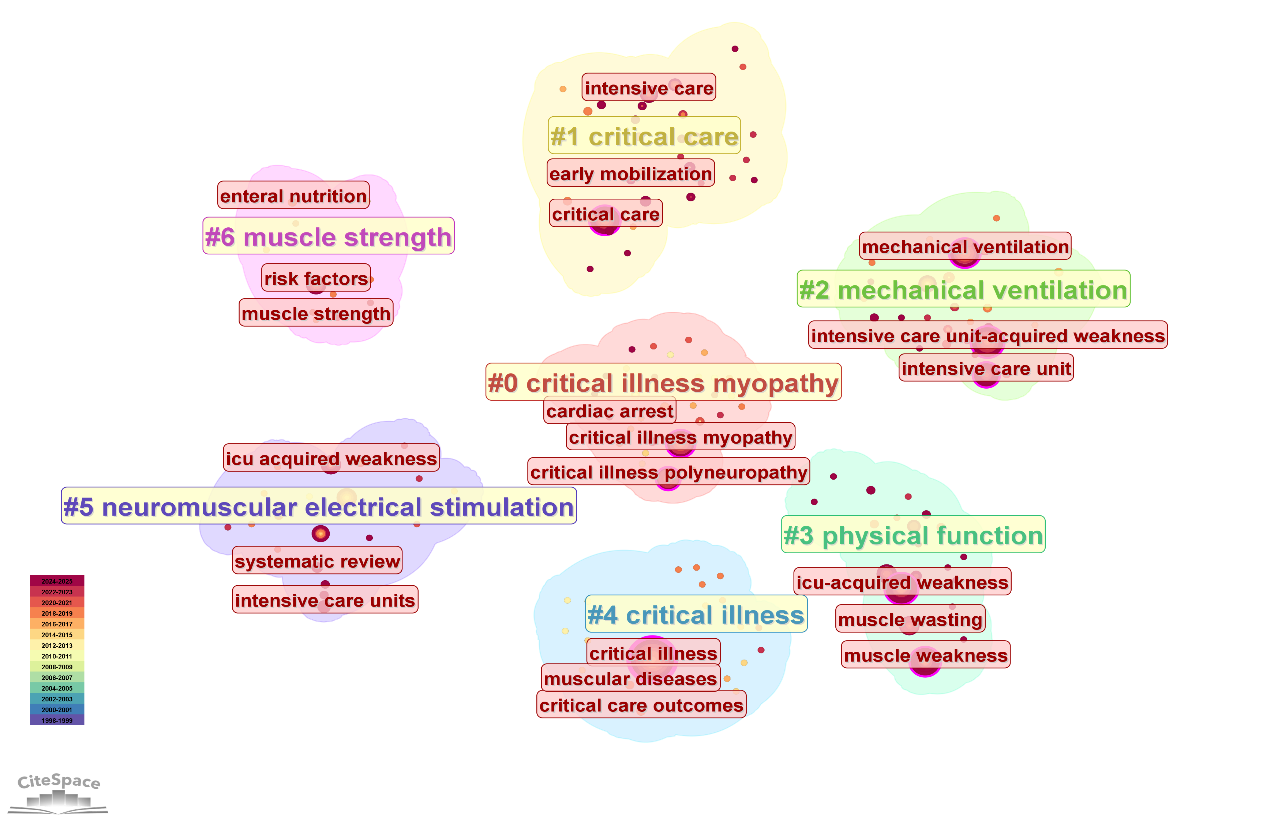


Figure S3: Keyword co-occurrence clusters in ICU-AW research in PubMed.
